# Comparative Effectiveness of Natural Versus Synthetic Biodegradable Scaffolds in Soft Tissue Reconstruction: A Systematic Review and Meta-Analysis

**DOI:** 10.7759/cureus.107399

**Published:** 2026-04-20

**Authors:** Olurotimi J Badero, Sandra Bakare, Chukwumbana Faith Okirie, Mohammad Al-Awadh

**Affiliations:** 1 Interventional Cardiology, Iwosan Lagoon Hospital, Lagos, NGA; 2 Interventional Cardiology, Division of Cardio-Nephrology, Cardiac Renal &amp; Vascular Associates, Jackson, USA; 3 General Medicine, Ternopil State Medical University, Ternopil, UKR; 4 Faculty of Medicine, Near East University, Nicosia, Cyprus; 5 General Surgery, University of Bahri, Khartoum, SDN

**Keywords:** biodegradable scaffolds, natural polymers, soft tissue reconstruction, synthetic polymers, tissue engineering

## Abstract

Biodegradable scaffolds are widely used in soft tissue reconstruction to support tissue regeneration and reduce long-term complications, yet the comparative clinical effectiveness of natural and synthetic scaffolds remains unclear. This systematic review and meta-analysis evaluated outcomes including recurrence rate, healing time, hospitalization duration, and complications. Following PRISMA guidelines and PROSPERO registration, PubMed, Scopus, and Web of Science were searched for studies published between January 2015 and January 2026. Randomized controlled trials and cohort studies involving human participants undergoing soft tissue reconstruction were included. Nine studies across multiple surgical fields were analyzed. Pooled results showed a significantly lower recurrence rate with natural scaffolds compared to synthetic scaffolds (RR 0.30, 95% CI 0.12 to 0.73, p = 0.008), with no heterogeneity (I² = 0%). Healing time did not differ significantly between groups (standard mean difference [SMD] -0.13, 95% CI -0.53 to 0.27, I² = 55%). Hospitalization time showed no significant difference but had high heterogeneity (SMD -0.76, 95% CI -0.06 to 1.57, I² = 89%). Complication rates favored natural scaffolds but were not statistically significant (RR 0.69, 95% CI 0.33 to 1.45, I² = 60%). Sensitivity analysis confirmed stability of findings. Natural scaffolds reduce recurrence and show a favorable safety trend, while healing and hospitalization outcomes depend more on surgical context than scaffold type.

## Introduction and background

Biodegradable materials are biomaterials designed to support cell adhesion, proliferation, and tissue regeneration, while gradually degrading into the cells [1]. Recently, biodegradable scaffolds have gained popularity among scientists and physicians as a better solution for soft tissue reconstruction. This is mainly due to the need for materials that will not only provide adequate structural support but also degrade in the host tissue without long-term complications. Soft tissue reconstruction is required following trauma, disease, or surgical resection to restore functional integrity, as well as to achieve aesthetic outcomes and reduce complications such as infection, fibrosis, or graft failure [2].

In clinical practice, commonly used scaffold materials can be classified into natural and synthetic scaffolds. Natural scaffolds closely mimic the extracellular matrix and are derived from biological sources (e.g., collagen, chitosan, alginate). This makes them the scaffold choice for regenerative applications where biological interactions are necessary, though their use may be limited due to variability in structure and reduced mechanical strength [3]. Synthetic scaffolds, on the other hand, are engineered polymers such as polylactic acid (PLA), polyglycolic acid (PGA), poly(lactic-co-glycolic acid) (PLGA), and polycaprolactone (PCL). They have better controlled mechanical strength and degradation kinetics. The disadvantage with these synthetic scaffolds is their lack of biological signals that are necessary for appropriate tissue regeneration and integration. Both scaffold types must show the required mechanical strength, porosity, biocompatibility, and controlled degradation kinetics to function properly in tissue [4].

Despite growing clinical use, direct comparison between natural and synthetic scaffolds in humans remains limited. Available literature cannot be directly applied into clinical practice because they are mostly based on in vitro experiments or animal studies. The relative advantage of each scaffold type in respect to healing, complications, and cost-effectiveness remains unclear, with existing literature reporting heterogeneous outcomes [5].

The aim of this systematic review and meta-analysis is to compare the clinical effectiveness of natural and synthetic biodegradable outcomes. The review will evaluate the comparison while focusing on key clinical outcomes, including complication rates, recurrence, healing time, and duration of hospitalization. This will provide a structured comparison of both scaffolds and guide physicians in optimal material selection in reconstructive practice.

## Review

Methodology

Inclusion Criteria

Studies were included if they met the following criteria:

Population: human participants of any age undergoing soft tissue reconstruction, including procedures involving skin, tendon, and muscle

Intervention: natural biodegradable scaffolds, including collagen, chitosan, and alginate

Comparator: synthetic biodegradable scaffolds, including PGA, PLGA, and polyacetic acid

Outcomes: complications, recurrence rate, healing time, and hospitalization tme

Study designs: randomized controlled trials and cohort studies

Studies that reported at least one of the clinically required outcomes including adverse effects

Studies from any location

Studies published in English

Exclusion Criteria

Studies that did not include both natural and synthetic scaffolds were not included in the study.

Additionally, exclusion criteria included animal models (in vivo and ex vivo), human ex vivo studies, previous systematic reviews and/or meta-analyses, case series, case reports, expert opinions, and unpublished studies (Table 1).

**Table 1:** Eligibility criteria for study selection based on the PICOS framework The table provides a summary of predefined inclusion and exclusion criteria used for study selection in this systematic review PGA, polyglycolic acid; PICOS, Population, Intervention, Comparator, Outcome, Study design; PLGA, poly(lactic-co-glycolic acid); RCT, randomized controlled trial

Category	Inclusion Criteria	Exclusion Criteria
Population	Human participants (any age) undergoing soft tissue reconstruction (skin, tendon, or muscle)	Animal models (In vivo and ex vivo), human ex vivo studies
Intervention	Natural biodegradable scaffolds (e.g., collagen, chitosan, alginate)	Non-biodegradable materials; scaffolds not derived from natural sources
Comparator	Synthetic biodegradable scaffolds (e.g., PGA, PLGA, polylactic acid)	No comparator or non-synthetic scaffolds
Outcomes	Complications, recurrence rate, healing time, and hospitalization time	Any study without the included outcomes
Study design	RCTs, and cohort studies	Systematic reviews, meta-analyses, case series, case reports, expert opinions, and unpublished data
Language and scope	Studies published in English; any geographical location	Non-English publications; abstracts only or gray literature
Reporting	Studies that report at least one of the clinically required outcomes including adverse effects	Studies that do not report any of the required outcome

Registration and Approval

This systematic review and meta-analysis were created in accordance with the Preferred Reporting Items for Systematic Reviews and Meta-Analyses (PRISMA) guidelines [6] to ensure transparency and methodical accuracy. Review protocol was registered in Prospero with registration number CRD420251090042 [7]. All methodological decisions, including the eligibility criteria, search strategy, outcomes of interest, and planned analytical approaches, were defined before initiating the review process.

Search Strategy

The search strategy targeted published randomized controlled trials and cohort studies that indicated both the natural and synthetic scaffolds in soft tissue reconstruction. The search incorporated both Medical Subject Headings (MeSH) and free-text terms and adhered to the Population, Intervention, Comparator, Outcome, Study design (PICOS) framework. Terms related to biodegradable scaffold, soft tissue reconstruction, natural scaffolds, synthetic scaffolds, PGA, collagen, PLGA, skin regeneration, and tissue engineering were combined using Boolean strings. The search strategy is presented in Table 2.

**Table 2:** Search strategy PubMed, Scopus, and Web of Science MESH search and Boolean string

Number	Search Terms
#1	("Biodegradable Scaffold"[Title/Abstract] OR "Bioabsorbable Scaffold"[Title/Abstract] OR "Resorbable Scaffold"[Title/Abstract] OR "Natural Scaffold"[Title/Abstract] OR "Synthetic Scaffold"[Title/Abstract] OR "Tissue Scaffold*"[Title/Abstract] OR "Polyglycolic acid"[Title/Abstract] OR "PGA"[Title/Abstract] OR "PLGA"[Title/Abstract] OR "Polyacetic acid"[Title/Abstract])
#2	("Soft tissue reconstruction"[Title/Abstract] OR "Tissue repair"[Title/Abstract] OR "Tendon repair"[Title/Abstract] OR "Skin regeneration"[Title/Abstract] OR "Muscle regeneration"[Title/Abstract] OR "Tissue Engineering"[MeSH Terms] OR "Tissue Engineering"[Title/Abstract])
#3	#1 and #2
#4	("Biodegradation"[Title/Abstract] OR "Biocompatible materials"[Title/Abstract])
#5	#3 and #4
Filter	English
Date	January 1, 2005, to January 9, 2026.

Data Extraction and Management

Data were extracted from the included studies by two authors to eliminate inaccurate records. Information extracted included study (author, year, country), detailed study design, sample size and population, intervention (natural scaffold), comparator (synthetic scaffold), follow-up duration, primary outcomes and key results, and secondary outcomes. Outcome data were extracted with corresponding 95% confidence intervals. Dichotomous data included the number of responders contributing to the counts of adverse effects such as complications and recurrence rate. Continuous data included the mean, standard deviation, and total number of patients, including outcomes such as hospitalization length and healing time. A meta-analysis was synthesized to highlight the trends in the efficacy, safety, and clinical applicability of the interventions.

Quality Assessment

We assessed the risk of bias for each included study using the appropriate tools according to the study design. Randomized trials were assessed using the Cochrane Risk of Bias tool RoB 2 [8], while non-randomized studies were assessed using ROBINS-I [9].

Two reviewers independently assessed each study blindly. Each study was rated as low risk, some concerns, or high risk based on the criteria of the tool. Disagreements were resolved by discussion and consensus, with a third reviewer in parallel. We interpreted evidence strength and avoided incorrect quantitative analysis where bias risk threatened the validity of results.

Outcome Measure

The primary outcome in this meta-analysis includes hospitalization time (total duration of stay in the hospital), recurrence rate (the measure of the scaffolds to inhibit the growth of the soft tissue), and healing time (this is the total duration of the initial surgery implantation point and when the scaffold has been reabsorbed or replaced). Secondary outcomes include complications (an adverse effect that occurred after implantation of the scaffolds). All efficacy outcome were extracted as reported by the original study authors.

Statistical Analysis

All statistical analyses were conducted using the tool Review Manager (RevMan). Continuous outcomes such as hospitalization length and healing time with corresponding 95% confidence intervals were extracted from published reports. Dichotomous outcomes such as complications and recurrence risk ratios and 95% confidence intervals were extracted from the published reports.

To account for identified heterogeneity across trials, a random-effects model (DerSimonian-Laird method) was employed. Heterogeneity was quantified using the chi-square test and the I² statistic, with interpretation based on Cochrane Handbook thresholds. Tau² was also reviewed to describe between-study variance. To evaluate the robustness of the findings, a sensitivity analysis was performed by re-analyzing the pooled dataset using a fixed-effect model. This allowed for an assessment of whether the overall treatment effect was influenced by the specific statistical model used. Data synthesis was performed using the inverse variance method. Continuous outcomes were expressed as mean differences or standardized mean differences (SMDs), while dichotomous outcomes were reported as risk ratios (RR), all presented with 95% confidence intervals.

RevMan was used to analyze publication bias using the funnel plots. Due to software limitations, formal statistical tests for small-study effects (e.g., Egger’s regression or Begg’s test) were not conducted. The certainty of the evidence for each outcome was subsequently evaluated considering the risk of bias, sensitivity, heterogeneity, and publication bias.

Ethical Considerations

As this was a systematic review of published literature, no ethical approval was required. The study was conducted in accordance with the Declaration of Helsinki. All tools used in the study, such as PRISMA [6], PROSPERO [7], Rayyan [10], Rob 2 [8], and ROBINS-I (assessing risk of bias in non-randomized studies of interventions) [9] were free to use.

Results

Study Characteristics

**Figure 1:**
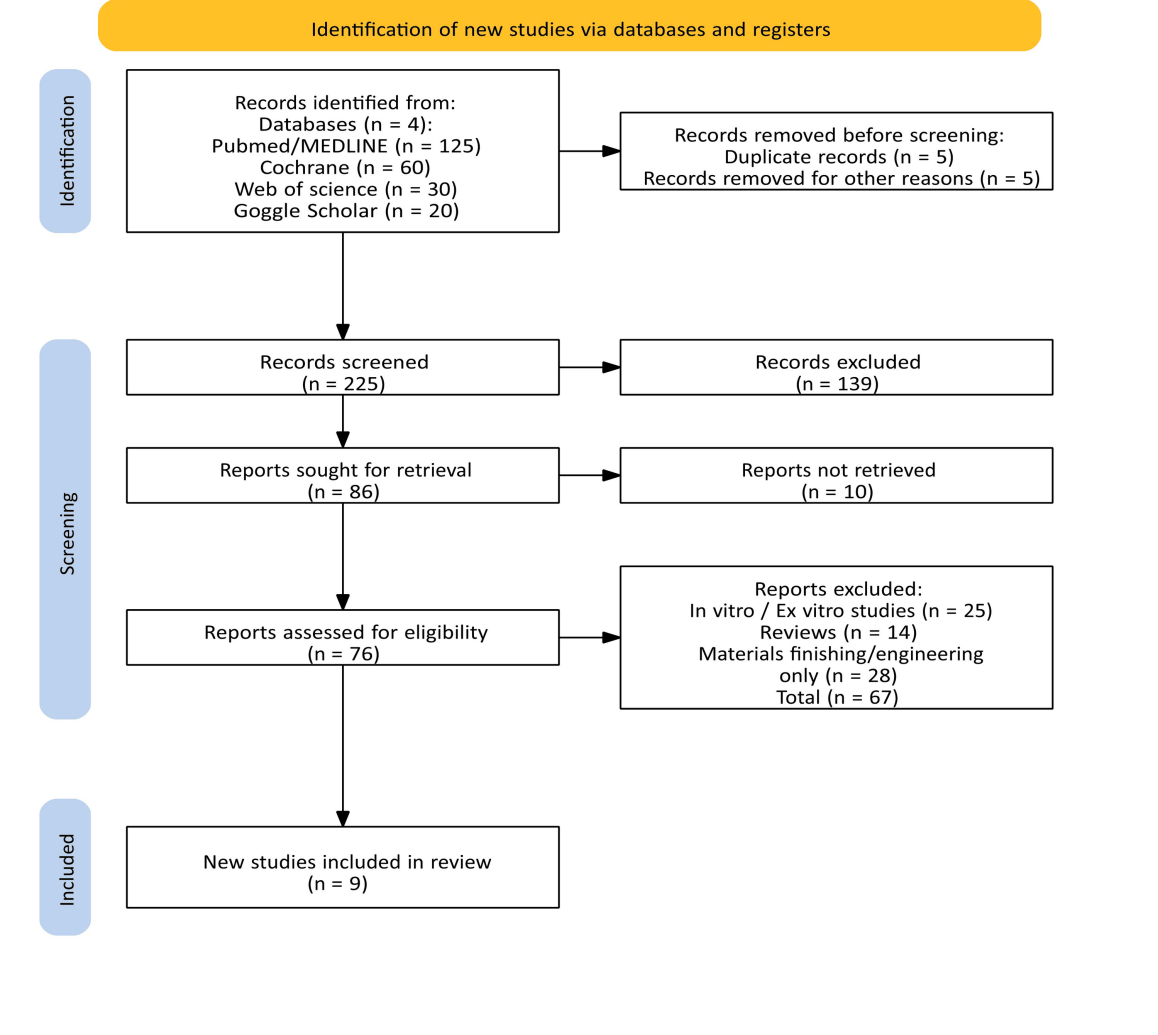
PRISMA flowchart showing the process of study selection [6]

We identified 235 records from database searching across PubMed/MEDLINE (n = 125), Scopus (n = 60), Web of Science (n = 30), and Google Scholar (n = 20), although Google Scholar lacks controlled vocabulary, inconsistent indexing, and limited transparency of its search algorithm focusing on date ranging from January 1, 2005, to January 9, 2026. Consistent with PRISMA 2020 [6] reporting, we tracked records through identification, screening, eligibility assessment, and inclusion criteria. Before screening, we removed 10 records, including five duplicates and five removed for other reasons, leaving 225 records for title and abstract screening. We excluded 139 records, and 10 reports could not be retrieved. Full text of 76 studies was assessed for eligibility, of which a total of 67 studies were excluded as they were vitro or ex vivo studies (n = 25), reviews (n = 14), and studies focused on materials finishing or engineering only (n = 28). A total of nine studies met the eligibility criteria using the Rayyan tool [10] (Figure 1). For record maintaining, differences and total number were settled through discussions held with a third reviewer. A summary of study characteristics and outcomes is presented in Table 3, and a summary of key quantitative outcomes is presented in Table 4.

**Table 3:** Summary of the characteristics and outcomes of the studies ADM, acellular dermal matrix; Ag, silver; CMC, carboxymethylcellulose; GBR, guided bone regeneration; GERD, gastroesophageal reflux disease; P4HB, poly-4-hydroxybutyrate; PCL, polycaprolactone; PEG, polyethylene glycol; PET, polyethylene terephthalate; PGA, polyglycolic acid; PLGA, poly(lactic-co-glycolic acid); RCT, randomized controlled trial; TBSA, total body surface area; TMC, trimethylene carbonate; TRM, tissue regeneration membrane

Study (Author, Year, Country)	Detailed Study Design	Sample Size and Population	Intervention (Natural Scaffold)	Comparator (Synthetic Scaffold)	Follow-Up Duration	Primary Outcomes and Key Results	Secondary Outcome
Gee Kee et al., 2015, Australia [11]	RCT (3-arm), single-center (burn center)	N=96 children with acute partial-thickness burns (≤10% TBSA)	Mepilex Ag dressing (Ag-impregnated foam) – natural (Ag)	Acticoat (nanocrystalline Ag mesh) ± Mepitel (silicone layer) – synthetic	Until full re-epithelialization (~2 weeks)	Mepilex Ag achieved significantly faster wound healing and less pain vs Acticoat-based dressings. Mepilex Ag reduced time to full re-epithelialization by ~33–40% compared to Acticoat/Acticoat+Mepitel. It also resulted in lower pain scores during dressing changes (vs Acticoat). No infections occurred in any group.	Pain scores were similar between Mepilex Ag and Acticoat+Mepitel groups at all time points. All three Ag dressings were safe and well-tolerated, with no significant differences in distress during dressing changes.
Harma et al., 2020, Turkey [12]	RCT, single-blinded (burn unit)	N=50 children (small/mid-size partial-thickness burns <30% TBSA)	Calcium alginate dressings (with or without zinc) – natural polysaccharide. Ag dressings (CMC hydrofiber-Ag or PET fiber-Ag) – hybrid	Nitrofurazone (0.2%) gauze dressing – standard control (no advanced scaffold)	21 days (biopsy at day 21)	No significant differences in key histological healing parameters were found among the advanced dressing groups (Ag-impregnated vs alginate). All advanced dressings showed superior healing quality compared to the plain gauze control. The Ag, calcium, and zinc-containing dressings each promoted robust granulation and re-epithelialization.	The dressings containing Ag or alginate had similar beneficial effects on wound healing (collagen deposition, fibroblast infiltration) in non-infected burns. Traditional antibacterial gauze was less effective. This suggests that Ag dressings are not uniquely superior–natural alginate scaffolds can be equally effective in pediatric burn care.
Buell et al., 2021, USA [13]	Retrospective cohort study (single institution)	N=73 patients (contaminated complex abdominal wall reconstructions)	Porcine biologic mesh (cadaveric dermis) – natural scaffold	Poly-4-hydroxybutyrate (P4HB) mesh (Phasix® fully absorbable) – synthetic	5 years	Over the 5-year follow-up, the synthetic P4HB mesh had a significantly lower hernia recurrence rate (12.9%) compared to the biologic mesh group (38.1%, P=0.017). This indicates superior durability of the synthetic scaffold in contaminated abdominal wall repairs.	No significant differences were noted in wound morbidity: infection and fistula rates were low and comparable between groups. The study suggests that absorbable synthetic mesh can achieve better long-term abdominal wall integrity than a biologic, without added complications, in contaminated fields.
Charleux-Muller et al., 2022, France [14]	Before–after cohort + decision-model cost-effectiveness	N=94 adults with contaminated ventral hernia repairs (single-center)	Biosynthetic absorbable mesh (e.g. PGA:TMC or P4HB polymer mesh) – synthetic (absorbable)	Biologic mesh (porcine acellular dermis) – natural collagen	≤1 year (perioperative outcomes; modeled long-term)	Use of a biosynthetic mesh led to fewer serious complications (21% vs 33% with biologic mesh) and lower total costs. A cost savings of ~$5,146 per case was estimated with biosynthetic scaffolds. Fewer wound failures/infections were observed in the synthetic group (no statistical comparison reported).	Long-term hernia recurrence rates were not reported in detail (study focused on short-term outcomes and cost). Authors conclude biosynthetic meshes can be a cost-effective alternative to expensive biologic meshes in contaminated fields, with comparable or better safety.
Liu et al., 2025, China [15]	RCT, prospective, single-center	N=124 patients (laparoscopic hiatal hernia repair)	Biologic mesh for crural reinforcement (non-crosslinked porcine dermis) – natural	Polypropylene mesh with anti-adhesion barrier – synthetic	18 months (with 6, 12, 18 months follow-up)	Hiatal hernia recurrence was low in both groups and not significantly different: 9.3% with biologic vs 1.9% with synthetic mesh at 18 months (P=0.225). Both groups saw substantial symptom improvement (GERD relief) and similar quality-of-life gains postoperatively. No mesh migrations or erosions occurred.	Early satiety symptoms differed slightly: at 6 months, the biologic mesh group reported less satiety (better) than synthetic mesh, and by 18 months, the synthetic group reported less satiety (better). These transient differences aside, both meshes were equally safe and effective, with no significant differences in other complications or overall patient satisfaction.
Mookerjee et al., 2023, USA [16]	Retrospective cohort (single institution)	N=46 patients (prepectoral breast reconstructions: 12 with Vicryl mesh vs 34 with ADM)	Vicryl (polyglactin 910) mesh wrap (knitted absorbable mesh) – synthetic	ADM (AlloDerm® or FlexHD®) – natural collagen	~3 months (short-term outcomes)	The Vicryl mesh prepectoral technique demonstrated comparable clinical outcomes to ADM. Overall complication rates were low and not significantly different (Vicryl: 2 infections, 1 skin necrosis; ADM: similar incidence). No implant losses occurred in either group.	The Vicryl mesh method markedly reduced operative time per breast (~35.7 min vs 68.0 min with ADM, P<0.01) and cut material costs by about $8,273 per breast. Patient outcomes (early healing and complication profile) were equivalent between groups, indicating that the synthetic Vicryl scaffold is a safe, much more cost-effective alternative to biologic ADMs in implant breast reconstruction.
Kusirisin et al., 2023, Thailand [17]	RCT, single-blinded (dentistry)	N=24 patients (GBR with dental implants)	Collagen membrane (Cytoplast™ RTM collagen) – natural (bovine collagen)	Bilayer PCL membrane (electrospun PCL) – synthetic	12 months	Both membranes yielded comparable bone regeneration outcomes at the 1-year follow-up. Radiographic ridge/bone thickness preservation and implant success rates did not significantly differ between the PCL membrane and the collagen membrane (noninferiority demonstrated).	No differences in postoperative infection or healing complications were observed between groups. Both the synthetic PCL scaffold and the natural collagen performed similarly in clinical and radiographic outcomes, indicating that the new PCL membrane is as safe and effective as the standard collagen membrane.
Jung et al., 2009, Switzerland [18]	RCT (split-mouth design)	N=10 patients (20 dental implant sites with bone defects)	None (standard guided bone graft alone) – control group received a collagen membrane	PEG–PLGA hydrogel membrane (experimental resorbable synthetic)	~6 months (re-entry for implant)	Bone regeneration success was achieved at all sites with both membranes. The quantity and quality of new bone at 6 months were similar between the synthetic PEG–PLGA hydrogel membrane and the conventional collagen membrane (no significant difference reported).	No membrane-related adverse events occurred. Histology showed well-vascularized new bone in both groups. This pilot trial indicates the synthetic degradable membrane can achieve equivalent GBR outcomes to a natural collagen membrane in dental implant sites.
Tang et al., 2025, China [19]	RCT, single-blind, noninferiority trial	N=48 patients (oral soft-tissue defects after lesion excision)	Gelatin–PCL composite membrane (electrospun hybrid scaffold) – natural+synthetic	Collagen membrane (bovine type I collagen) – natural	3 months	Complete mucosal healing (grade A) was achieved in 100% of cases by 1 month in both the gelatin–PCL TRM group and the collagen membrane group. The experimental scaffold met noninferiority criteria for healing rate (no worse outcomes than collagen). No differences were found in wound contraction or time-to-healing between groups.	Instrument handling and patient satisfaction were similar for both groups. Adverse event rates did not differ significantly (no serious scaffold-related events). Thus, the gelatin–PCL TRM proved equally safe and effective as the standard collagen membrane for oral soft tissue reconstruction.

**Table 4:** Summary of key quantitative outcomes Comparative presentation of quantitative outcome measures across burn surgery, abdominal wall and hernia repair, breast reconstruction, and dental applications. Outcomes include recurrence rates, complication rates, healing times, and hospitalization time. P4HB, poly-4-hydroxybutyrate; PCL, polycaprolactone; PEG, polyethylene glycol; PGA, polyglycolic acid; PLGA, poly(lactic-co-glycolic acid)

Surgery Type	Study	Patients (n)	Natural Scaffold Evaluated	Synthetic Scaffold Evaluated	Primary Category	Main Result	P-value
Burns (children)	Gee Kee et al. (2015) [11]	96	Silver-impregnated foam	Nanocrystalline silver mesh	Natural vs synthetic	Natural healed 33–40% faster	NR
	Harma et al. (2020) [12]	50	Calcium alginate ± zinc	Silver fiber dressings	Natural vs synthetic	All advanced dressings performed equally	NS
Abdominal/hernia	Buell et al. (2021) [13]	73	Porcine biologic mesh	P4HB absorbable synthetic	Natural vs synthetic	5-year recurrence: 12.9% synthetic vs 38.1% natural	p=0.017
	Charleux-Muller et al. (2022) [14]	94	Porcine biologic mesh	Biosynthetic absorbable (PGA:TMC/P4HB)	Natural vs biosynthetic	Lower complications (21%) and saved ~$5,146/patient with synthetic	NR
	Liu et al. (2025) [15]	124	Porcine biologic mesh	Polypropylene mesh (anti-adhesion)	Natural vs synthetic	18-month recurrence: 1.9% synthetic vs 9.3% natural	p=0.225
Breast surgery	Mookerjee et al. (2023) [16]	46	ADM	Vicryl (polyglactin 910)	Natural vs synthetic	Similar outcomes; synthetic saved ~$8,273/patient	NS
Dental/oral	Kusirisin et al. (2023) [17]	24	Bovine collagen	PCL	Natural vs synthetic	Bone regeneration comparable at 12 months	NS
	Jung et al. (2009) [18]	10	Collagen membrane	PEG–PLGA hydrogel	Natural vs synthetic	Bone quality/quantity similar at 6 months	NS
	Tang et al. (2025) [19]	48	Collagen membrane	Gelatin–PCL composite	Natural vs hybrid	Complete healing: 100% in both groups by 1 month	NS

Evaluation of risk of bias

**Figure 2:**
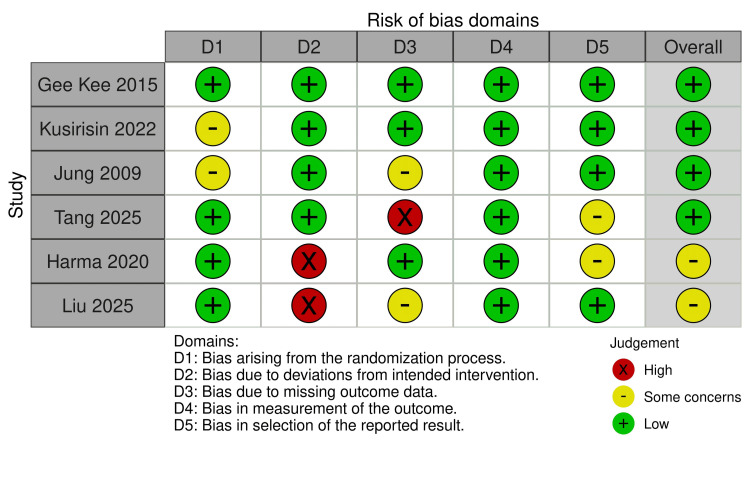
Risk of bias across randomized controlled trials Aggregate proportion of studies judged as low, some concerns, or high risk across the Cochrane RoB domains. Studies: Gee Kee et al. [11], Kusirisin et al. [17], Jung et al. [18], Tang et al. [19], Harma et al. [12], Liu et al. [15]

**Figure 3:**
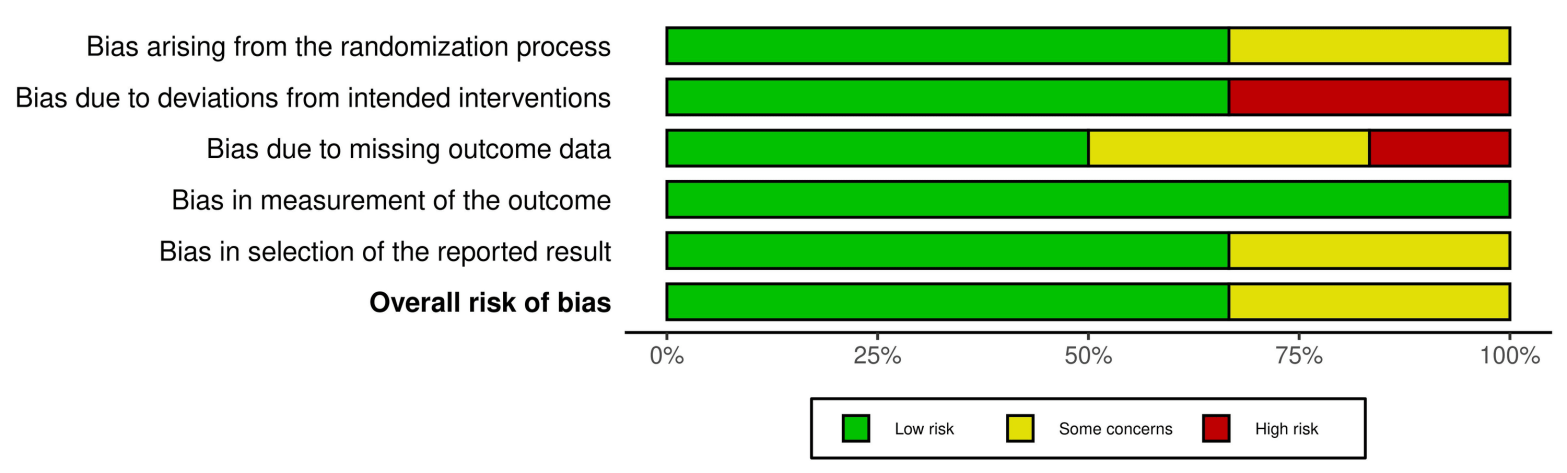
Summary of risk of bias across all domains Aggregate proportion of studies judged as low, some concerns, or high risk across the Cochrane RoB domains

**Figure 4:**
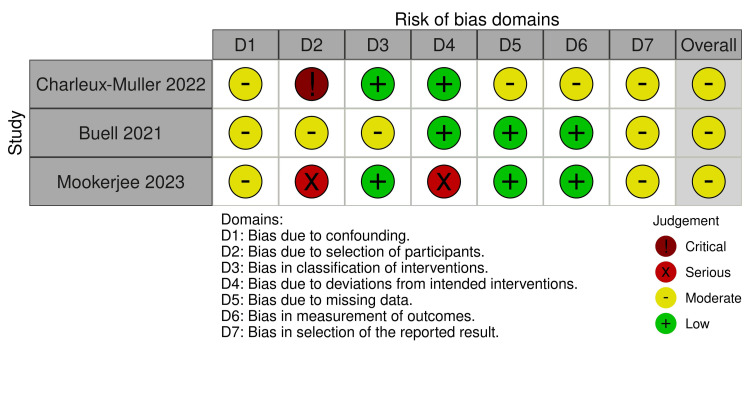
Summary of risk of bias across all cohort studies Aggregate proportion of studies judged as low, some concerns, or high risk across the Cochrane ROBINS-I domains. Studies: Charleux-Muller et al. [14], Buell et al. [13], Mookerjee et al. [16]

**Figure 5:**
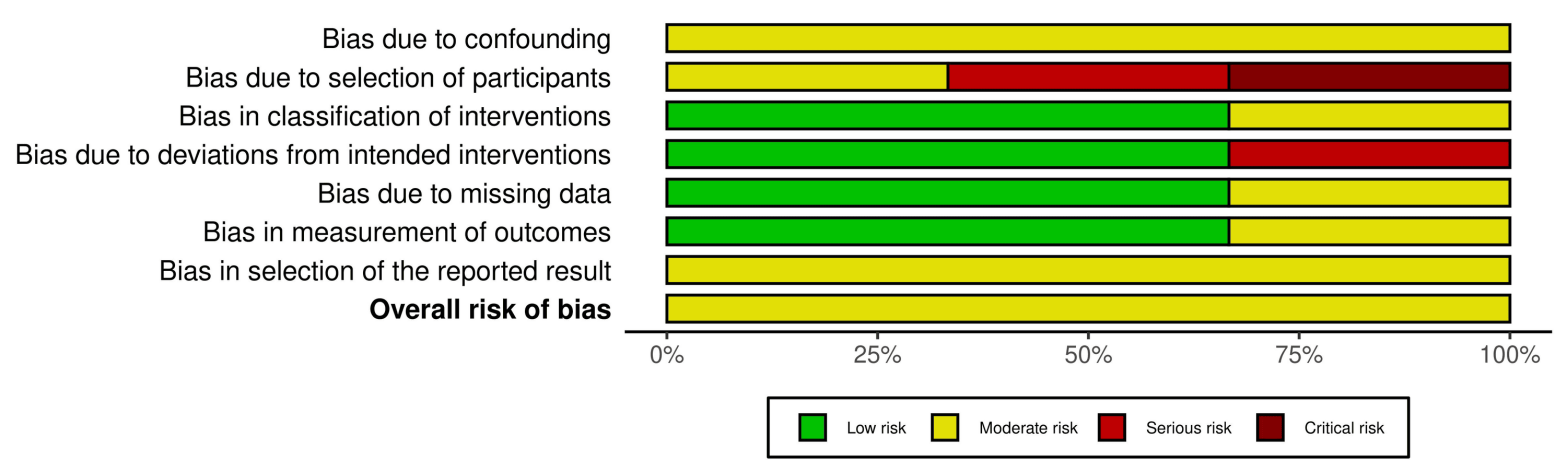
Summary of risk of bias across all cohort domains Aggregate proportion of studies judged as low, some concerns, or high risk across the Cochrane ROBINS-I domains

The quality of the methodology of all the included nine studies was assessed using the Rob2 for the six randomized controlled trials studies and ROBINS-1 for the three cohort studies, with the result summarized in Figures 2-5. Across the RCT trials, four studies showed a clear description of randomization and non-inferiority margin, which resulted in a low risk of selection bias [11,17-19], while two studies lacked allocation concealment and clear blinding details and had a mix of objective and subjective outcomes, resulting in some concerns about the risk of bias [12,15]. The three cohort studies had a moderate risk of bias, as it presented with clear statistical adjustment, potential incomplete long-term follow-up, and a small sample with unequal groups [13,14,16]. Publication bias assessment indicated a balanced funnel plot and a low likelihood of publication bias, and the close cluster near zero suggested little or no overall effect differences between the study groups (Appendix A).

Primary outcomes

Recurrence

**Figure 6:**
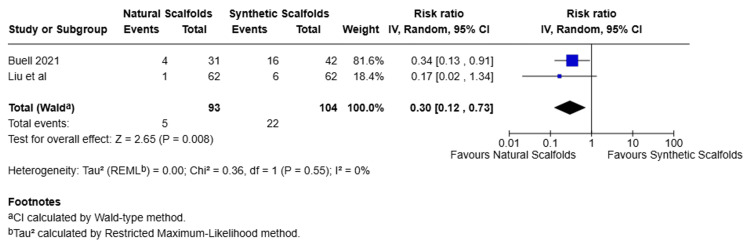
Forest plot for recurrence rate (randomized effects) Forest plot comparing recurrence rates between natural scaffolds and synthetic scaffolds. Studies: Buell et al. [13], Liu et al. [15] CI, confidence interval; df, degrees of freedom; I², inconsistency index; IV, inverse variance; P, p-value; Z, Z statistic for overall effect

Two studies were pooled to compare the recurrence rate between natural scaffolds and synthetic scaffolds and showed that patients treated with natural scaffolds had a 70% lower risk of recurrence compared to those treated with synthetic scaffolds (RR 0.30, 95% CI 012- 0.73, p=0.008). No significant heterogeneity was observed between the studies (I2=0%, Chi2=0.36, p=0.55) (Figure 6).

Healing Time

**Figure 7:**
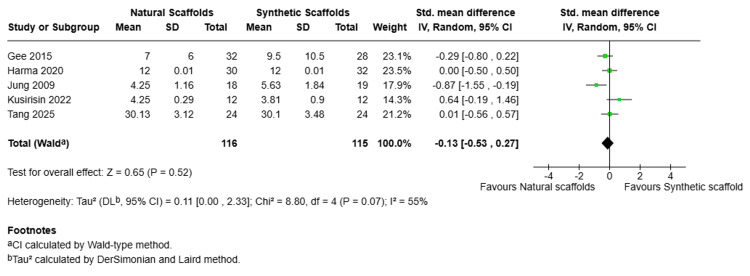
Forest plot for healing time (randomized effect) Forest plot comparing healing time between natural scaffolds and synthetic scaffolds. Studies: Gee Kee et al. [11], Harma et al. [12], Jung et al. [18], Kusirisin et al. [17], Tang et al. [19] CI, confidence interval; df, degrees of freedom; I², inconsistency index; IV, inverse variance; P, p-value; SD, standard deviation; Z, Z statistic for overall effect

Five studies were included in the pooled analysis to compare the total duration of the initial surgery implantation point and when the scaffold has been reabsorbed or replaced between the natural and synthetic scaffolds. As shown in Figure 7, the SDM was -0.13 (95% CI: -0.53, 0.27), indicating a slight but non-significant trend toward shorter healing times with natural scaffolds. Moderate heterogeneity (I2=55%, Chi2=8.80, p=0.07) was reported among the studies.

Hospitalization Time

**Figure 8:**
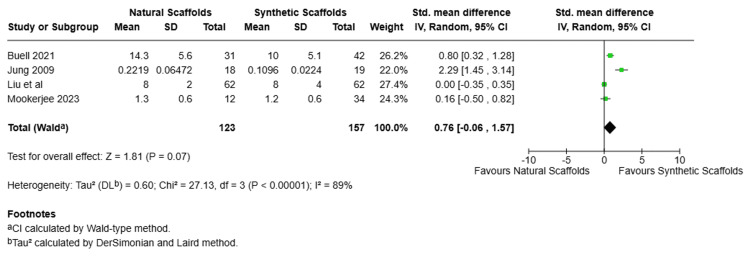
Forest plot for hospitalization time (randomized effect) Forest plot comparing hospitalization time between natural scaffolds and synthetic scaffolds. Studies: Buell et al. [13], Jung et al. [18], Liu et al. [15], Mookerjee et al. [16] CI, confidence interval; df, degrees of freedom; I², inconsistency index; IV, inverse variance; P, p-value; SD, standard deviation; Z, Z statistic for overall effect

Four studies were pooled to compare the duration of patient stay in the hospital after surgical procedure using natural scaffold or synthetic scaffold. As shown in Figure 8, SMD was -0.76, with 95% CI of -0.06, 1.57, suggesting that hospitalization is longer with natural scaffolds. High heterogeneity (I2=89%, Chi2=27.13, p=0.00001) and no statistical significance difference were reported among the studies.

Secondary outcomes

Complications

**Figure 9:**
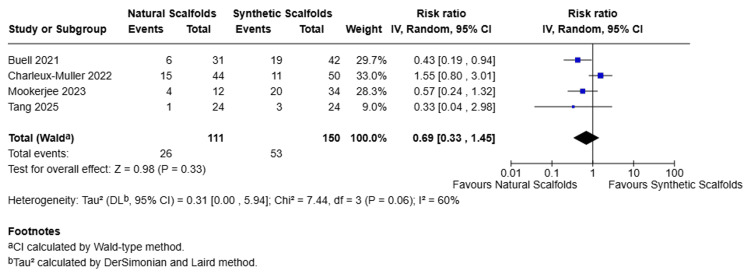
Forest plot for complications (randomized effects) Forest plot comparing complications between natural scaffolds and synthetic scaffolds. Studies: Buell et al. [13], Charleux-Muller et al. [14], Mookerjee et al. [16], Tang et al. [19] CI, confidence interval; df, degrees of freedom; I², inconsistency index; IV, inverse variance; P, p-value; SD, standard deviation; Z, Z statistic for overall effect

Four studies compared the complications of natural scaffolds against synthetic scaffolds. As shown in Figure 9, the pooled estimates suggest a trend towards fewer complications with natural scaffolds compared to the synthetic scaffolds (RR 0.69, 95% CI 0.33-1.45, p=0.44). Moderate heterogenicity (I2=60%, Chi2=7.44, p=0.06) was reported among the studies

Sensitivity Analysis

Fixed-effect models were used to evaluate the sensitivity of the pooled studies across outcomes (Appendix B-E). For healing time, the fixed-effect yielded the same pooled confidence interval ratio, with the point estimate barely increasing, suggesting that the difference between natural and synthetic scaffolds was consistent. Although the analysis of hospitalization time presented a lower SMD under the fixed-effect model, the overall interpretation stayed the same. For recurrence, there was no change in the RR, P-value, and the overall effect, showing strong stability across both models. For complication, the fixed effect remained on the left side of the null effect, which means that it is consistent with the random effect model, indicating stability that there are lesser complications with the natural scaffolds.

The fixed-effect model results indicate that the overall findings are robust and not materially influenced by the weighting structure or the level of heterogeneity across the statistical models. This detailed methodical evidence shows validity of the conclusion pooled form studied outcomes.

Discussion

This meta-analysis highlights the biologic advantage of soft tissue reconstruction for natural scaffold compared to the synthetic, especially in the prevention of recurrence rate, possibly due to its ability to undergo remodeling. This aligns with the conclusion that natural extracellular matrix reduces the response of a foreign body compared to the escalation that occurs when a synthetic polymer is used [20]. This suggests that the natural scaffold is more bio-friendly and aids in tissue integration. Although multiple types of scaffolds were included in the study, ranging from acellular dermal matrices and collagen-based graft for natural scaffold to PGA, PLA, and PCL for synthetic scaffold, results remain the same across board.

The studies included in this analysis investigated soft tissue reconstruction across various anatomical sites, including dental/oral tissues [17-19], pediatric wounds [11,12], abdominal hernias [13-15], and breast tissue [16]. This produced a high heterogeneity in the hospitalization time because the recovery time for an oral tissue is largely different from that of abdominal hernia repair or breast reconstruction. The sensitivity analysis showed that the significance of hospitalization data was volatile, supporting that the type of scaffold is a secondary factor to a specific surgical procedure or the location of the procedure, which determines both patients’ healing time and how long they will stay in the hospital.

While the overall safety profile remains complex, pooled data for complications numerically favored natural scaffolds; however, this difference did not reach statistical significance. Notably, sensitivity analysis revealed that one study [14] showed a significant difference favoring synthetic scaffolds, likely due to specific surgical techniques or the particular synthetic polymer used in that trial. This is consistent with evidence from two studies on ventral hernia repair, where synthetic meshes demonstrated greater durability and fewer complications, remaining the preferred option over natural scaffolds [21,22]. Meanwhile a study highlighted the importance of both and suggested that for bone regeneration, a combination of both scaffolds could create a combination of both biocompatibility and physiological compatibility [23]. One study noted that natural collagen matrices effectively promote adhesion by activating myofibroblasts and fibroblasts at the wound site, which triggers cytokine secretion and anti-inflammatory mediators more effectively than synthetic alternatives [24,25].

This study is limited due to the inadequate long-term follow-up of more than 24 months in most of the included studies, which may have limited the accurate data for recurrence or healing time. Furthermore, the "natural scaffold" category is broad, encompassing various porcine, bovine, and human-derived materials, which may have different immunogenic profiles. Due to a lack of sufficient data for other outcomes, the meta-analysis was limited to four primary metrics, although further outcomes may emerge as more studies become available.

The future of scaffold technology will likely focus on closing the identified gaps between material functionalities and patient-centered needs. This includes the development of three-generation membranes with bioactive layers for antimicrobial release and the use of CAD/CAM and 3D bioprinting to create patient-specific scaffolds that better mimic the complex geometry of human soft tissue. As clinical evidence continues to mount, the decision to use a natural or synthetic scaffold will be increasingly guided by evidence-based indications that prioritize not only the speed of healing but also the quality of life, cost of care, and long-term durability of the reconstruction.

## Conclusions

This systematic review and meta-analysis offers valuable insight into the complication, recurrence rate, healing, and hospitalization time of diverse types of natural and synthetic scaffolds in various soft tissue sites. The analysis strongly supports natural scaffolds as superior in preventing recurrence. While there is a trend toward fewer complications with certain scaffolds, no statistically significant differences were observed regarding healing duration or length of hospitalization. Surgeons should prioritize natural scaffolds when the primary clinical goal is long-term structural success.

## Figures and Tables

**Figure 10:**
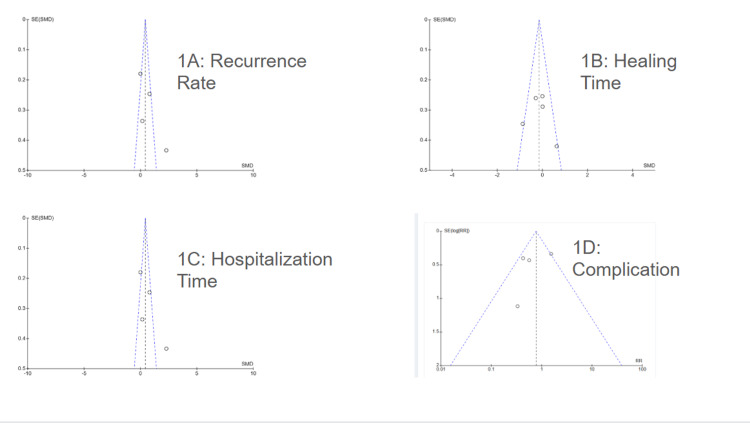
Funnel plots for publication bias Funnel plot assessing potential publication bias for recurrence rate, healing time, hospitalization time and complication outcomes. Standard error is plotted against log risk ratio. Symmetry indicates a low likelihood of small-study effects. SE, standard error; PFS, progression-free survival

**Figure 11:**
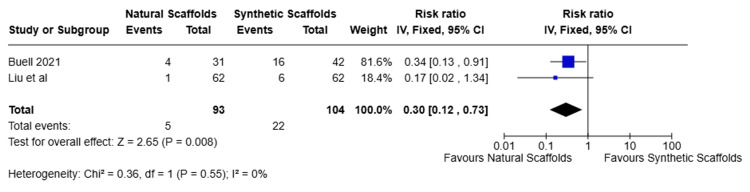
Forest plots (fixed effects) for sensitivity analysis Sensitivity analysis forest plot for recurrence rate using a fixed-effects model. Studies: Buell et al. [13], Liu et al. [15]

**Figure 12:**
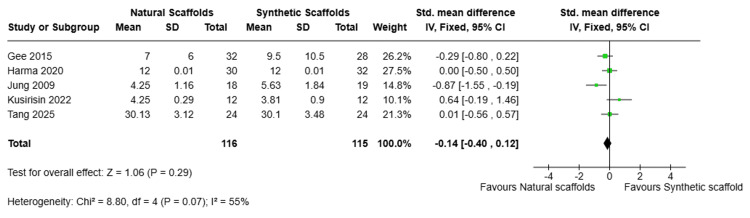
Forest plots (fixed effects) for sensitivity analysis Sensitivity analysis forest plot for healing time using a fixed-effects model. Studies: Gee Kee et al. [11], Harma et al. [12], Jung et al. [18], Kusirisin et al. [17], Tang et al. [19]

**Figure 13:**
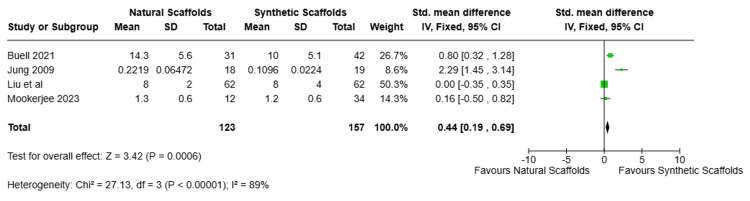
Forest plots (fixed effects) for sensitivity analysis Sensitivity analysis forest plot for hospitalization time using a fixed-effects model Studies: Buell et al. [13], Jung et al. [18], Liu et al. [15], Mookerjee et al. [16]

**Figure 14:**
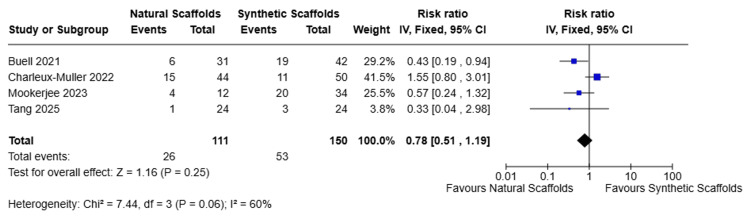
Forest plots (fixed effects) for sensitivity analysis Sensitivity analysis forest plot for complications using a fixed-effects model Studies: Buell et al. [13], Charleux-Muller et al. [14], Mookerjee et al. [16], Tang et al. [19]

## Appendices

Appendix A

Appendix B 

Appendix C 

Appendix D 

Appendix E 
